# A feasibility, randomised controlled trial of Club Connect: a group-based healthy brain ageing cognitive training program for older adults with major depression within an older people’s mental health service

**DOI:** 10.1186/s12888-023-05391-7

**Published:** 2024-03-18

**Authors:** Claudia Woolf, L. Kaplan, L. M. Norrie, D. Burke, M. Cunich, L. Mowszowski, S. L. Naismith

**Affiliations:** 1 Older People’s Mental Health Service, St Vincent’s Hospital, 390 Victoria St, Darlinghurst, Sydney, NSW 2010 Australia; 2 Healthy Brain Ageing Program, Brain and Mind Centre, The University of Sydney, Camperdown, NSW Australia; 3 Faculty of Science, School of Psychology, The University of Sydney, Camperdown, NSW Australia; 4 School of Psychiatry, University of New South Wales, Randwick, NSW Australia; 5 Discipline of Psychiatry, University of Notre Dame, Sydney, NSW Australia; 6 Department of Psychiatry, Trinity College, Dublin, Ireland; 7 Charles Perkins Centre, The Faculty of Medicine and Health (Central Clinical School), The University of Sydney, Camperdown, NSW Australia; 8 Sydney Health Economics Collaborative, Sydney Local Health District, Camperdown, NSW Australia

**Keywords:** Cognitive training, Cognitive remediation, Depression, Implementation, Feasibility, Older adults

## Abstract

**Background:**

Using the RE-AIM (reach, effectiveness, adoption, implementation, maintenance) framework, we outline steps taken to implement an evidence-based cognitive training program, Club Connect, in older adults with major depressive disorder in an Older People’s Mental Health Service in Sydney, Australia. The primary aim was to explore feasibility (or ‘reach’), tolerability (or ‘implementation’), and acceptability (or ‘adoption’). The secondary aim was to explore the most sensitive clinical outcomes and measurement tools (i.e. ‘effectiveness’) to inform a formal randomised controlled trial, and to explore the healthcare resources used (i.e. costs) to assist decision-making by health care managers and policy-makers in relation to future resource allocation.

**Methods:**

Using a single blinded feasibility design, 40 participants (mean age: 76.13 years, SD: 7.45, range: 65–95 years) were randomised to either (a) Club Connect, a 10-week group-based multifaceted program, comprising psychoeducation and computer-based cognitive training, or (b) a waitlist control group.

**Results:**

Implementing group-based cognitive training within a clinical setting was feasible, well tolerated and accepted by participants. Further, cognitive training, in comparison to the waiting list control, was associated with moderate to very large effect size improvements in depression, stress and inhibition (ηp^2^ = 0.115–0.209). We also found moderate effect size improvements on measures of daily functioning, wellbeing and cognitive flexibility. Small effect size improvements for other cognitive and psychosocial outcomes were also observed. The average cost per person participating in in the intervention was AU$607.50.

**Conclusions:**

Our findings support the feasibility of implementing group-based cognitive training into a specialised clinical (public health) setting. This trial was registered on the Australian and New Zealand Clinical Trial Registry (ACTRN12619000195156, 12/02/2019).

## Introduction

Major depressive disorder (MDD) is the leading cause of mental illness worldwide [1], and is associated with significant rates of morbidity and mortality [2]. While the hallmark symptoms of major depression are widely established to include persistent low mood and anhedonia, two thirds of all acutely unwell patients report concomitant neurocognitive impairment [3] which tends to be mildly to moderately severe in nature [4]. In the remission phase, neurocognitive impairment persists, even during states of euthymia, for as many as one third to one half of all previously depressed patients [5]. It is not surprising therefore that neurocognitive impairment is one of the strongest predictors of illness relapse or recurrence in MDD [6].

Given it is well recognised that neurocognitive impairment is unresponsive to pharmacological treatment [7]; constrains psychosocial and functional achievement at significant financial, social, familial and personal cost [8, 9]; adds to illness burden [10–14]; and is associated with mild cognitive impairment (MCI) (the prodromal condition to dementia) for 75% of older adults [15], we argue that symptomatic remission of low mood and anhedonia is insufficient as the primary goal of treatment in MDD. Neurocognitive impairment needs to be recognised as a primary intervention target in MDD, and with validated success in other chronic mental disorders, cognitive remediation is one avenue that holds promise.

Non-pharmacological interventions including cognitive remediation are designed to mitigate the implications of neurocognitive impairment. Three primary approaches have been identified: cognitive stimulation, cognitive training (CT) and cognitive rehabilitation (see [16] for an overview). Of these, CT is gaining prominence in light of evidence demonstrating that it has the potential to maintain and even, improve cognition, thereby improving psychosocial functioning. CT may be strategy-based [17], incorporating both internal and external compensatory cognitive strategies, or computer-based [17], typically incorporating drill-and-practice exercises targeting specific cognitive domains [16, 18, 19]. The underlying premise is that intensive cognitive exercises build or restore brain and cognitive integrity, promoting neuroplasticity and providing greater resilience against neuropathology, thereby maintaining function [20].

The literature supporting the efficacy of CT in various types of mental illness has been reported in several meta-analytic studies. In individuals with schizophrenia, consistent evidence from randomised controlled trials (RCT) demonstrates significant, durable improvements in cognition and daily functioning [21–25], and evidence is also emerging for the efficacy of CT for individual’s with first episode psychosis [26, 27]. There is also now emerging evidence for CT in individual’s with affective disorders. For late life depression (LLD) in particular, RCTs have demonstrated that computer-based CT [28, 29] and combined computer- and strategy-based CT [30] leads to improved performance on cognitive and affective outcomes.

While there is considerable enthusiasm for CT interventions amongst both consumers [31] and healthcare providers, CT is not routinely available within Australian mental health services [32] and little is known about how best to implement such programs in health care systems [33, 34]. In fact, one of the most critical issues in mental health services research is the gap between what is known about effective treatment and what is provided to and experienced by consumers as part of routine care in community settings [35]. This gap reflects, in large measure, a paucity of evidence about implementation, cost-effectiveness, and resource use (i.e. sustainability). Given this, researchers must recognise the need to not only evaluate clinical outcomes, but also to perform formative evaluations to assess and refine implementation.

While there are some studies of CT implementation in serious mental illness [36], including in psychosis and schizophrenia [34, 37, 38], and in depression [39, 40], as well as in MCI [41, 42], the evidence base is very limited with methodological shortcomings, and very few studies report comprehensively on key elements of research translation within a structured ‘implementation framework’. These frameworks are integral to distinguishing *implementation* effectiveness from *treatment* effectiveness, which is critical for translating interventions from research settings to public health settings [43]. In this regard, the RE-AIM framework outlines five steps to translate research into action to improve sustainable adoption and implementation of effective, generalisable, evidence-based interventions. These are defined as: (1) ‘Reach’ of the target population; (2) ‘Effectiveness’ of the intervention; (3) ‘Adoption’ by target staff, settings, or institutions; (4) ‘Implementation’ - consistency, costs and adaptions made during delivery; and (5) ‘Maintenance’ of intervention effects in individuals and settings over time [44]. Therefore, using the RE-AIM framework, we sought to conduct a feasibility RCT of a group-based CT program, ‘Club Connect’. Club Connect was established in 2015 as an attempt to translate the evidence-based Healthy Brain Ageing (HBA) program from a research setting (The University of Sydney) to a clinical setting (St Vincent’s Hospital, Sydney). Evaluations of the HBA program (which was first developed for help-seeking older adults ‘at risk’ of cognitive decline [30, 45]), have demonstrated improvements in memory and dementia literacy in those with late-life depression [30, 46]) and in memory, depressive symptoms, and sleep quality in those with mild cognitive impairment [45, 47], and improvements in memory in those with Parkinson’s disease [48]. Club Connect was piloted [49, 50] using a pre-post single arm study design at St Vincent’s Hospital, Sydney in 79 older adults with mild cognitive impairment and demonstrated that it was feasible to translate group-based CT to the clinical setting.

Therefore, we sought to explore feasibility or ‘Reach’ (of recruitment rates), tolerability or ‘Implementation’ (adherence to treatment protocol), and acceptability or ‘Adoption’ of Club Connect in older adults with major depression within a metropolitan Older People’s Mental Health Service. As secondary aims, we sought to explore the most sensitive clinical outcomes and measurement tools (i.e. ‘Effectiveness’) to inform a future full-scale trial, and to examine the resource use (costs) of delivering the intervention.

## Method

### Recruitment and setting

Participants were recruited from February to September 2019 from (a) Older People’s Mental Health or (b) Geriatric Medicine, both at St Vincent’s Hospital, Sydney, Australia, or (c) from the community in response to flyers in local general practitioner practices and advertising in a local newspaper. Referrals from within St Vincent’s Hospital or from local general practitioners were received at the weekly Older People’s Mental Health case conference meeting, where an individual’s suitability was discussed. Referrals from the community were received via telephone directly from individuals who self-referred; these referrals were also discussed at the weekly Older People’s Mental Health case conference meeting. If it was deemed that an individual may meet eligibility criteria, telephone screening (described below) was commenced.

In the absence of clear thresholds for feasibility of recruitment, we adopted a pragmatic, service-oriented approach where sample size was determined by the number of participants that was feasible to recruit within the designated timeframe (i.e. February-September 2019); this was based on pilot data [49, 50].

### Participants

Participants eligible for the study were:

- 65 years or older;
- with current depressive symptoms (as evidenced by ≥6 on the Geriatric Depression Scale 15-item (GDS-15) [51]) or history of a Major Depressive Episode within the last five years (assessed using the Mini International Neuropsychiatric Interview (MINI) [52]); and,
- those who were willing and able to commit to attending for the duration of the program, outside of unforeseen or unanticipated circumstances (e.g. illness).

Exclusion criteria were:

- an established diagnosis of dementia with impairment in activities of daily living (ADLs);
- a Mini Mental State Examination (MMSE) score < 24;
- severe major depression with impaired ADLs, or current harmful or dependent substance use (i.e. more than recommended daily intake based on national guidelines), or, current or history of, a non-affective psychiatric disorder (e.g. schizophrenia etc.) that could impede an individual’s ability to engage in group-based CT;
- electroconvulsive therapy within the three months prior to baseline assessment; and,
- insufficient English proficiency to participate in psychometric testing or in group-based CT.
       Participants engaging in other treatment for depression (i.e. non-pharmacological and pharmacological therapy) were included, although participants were required to be stabilised on their therapy for at least four weeks prior to baseline assessment.

### Procedure

As part of telephone screening, a checklist of questions addressing inclusion/exclusion criteria was administered as well as the GDS-15 [51] and the MINI [52]. If eligibility criteria were satisfied, a face-to-face baseline assessment was arranged within a fortnight of the intervention commencing. At baseline, eligibility was confirmed (including administration of the MMSE and in some cases, depending on time between screening and baseline, re-administration of the GDS-15 [51]), and informed written consent was obtained from all participants. In addition, all participants completed a standardised battery of neuropsychological tests and a range of measures assessing mood and psychosocial functioning, and all participants were reviewed by an Old Age Psychiatrist (DB, LN, YS) or their Psychiatry Registrar. These assessments were repeated within a fortnight following the completion of the ten-week intervention period.

### Design

**Fig. 1:**
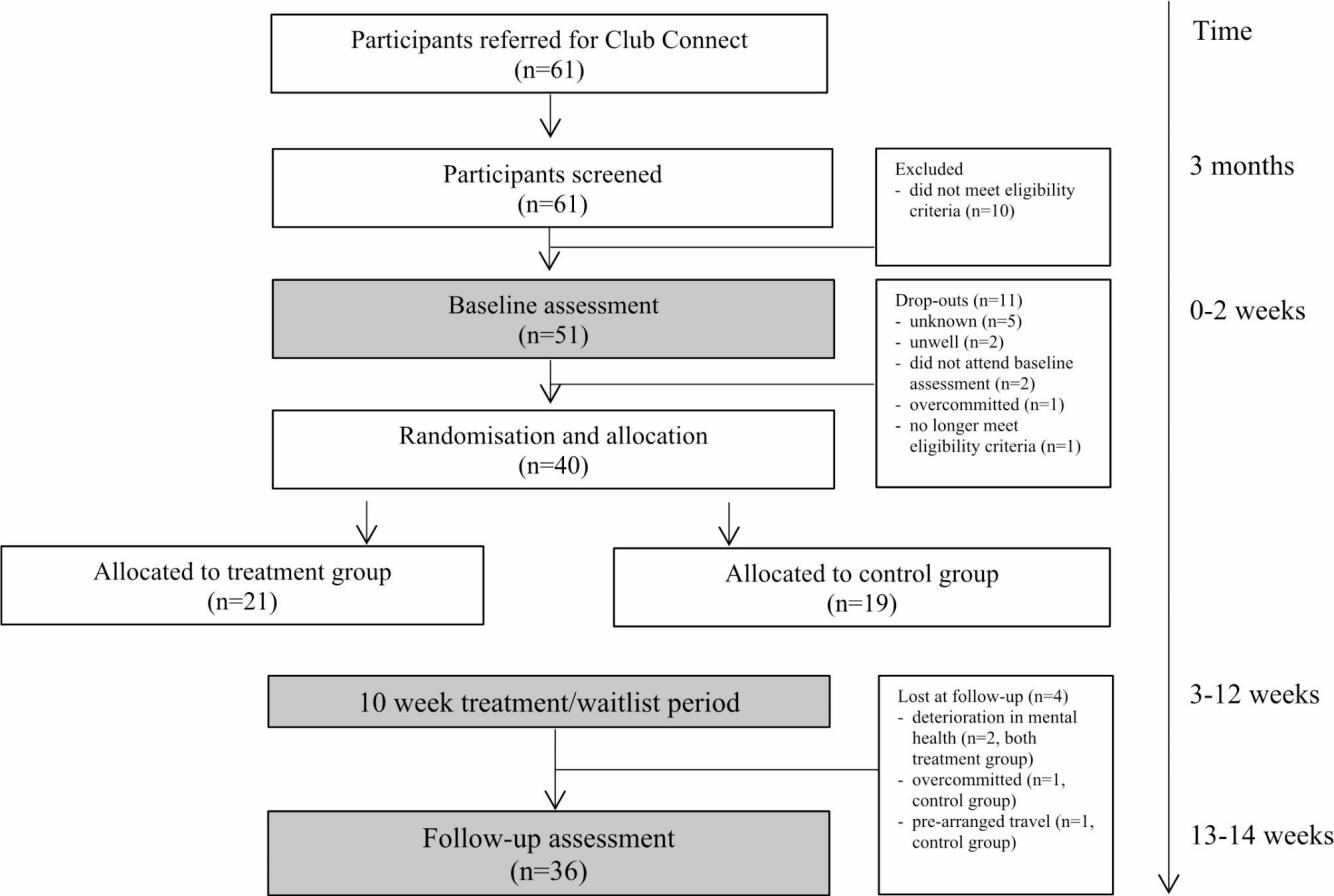
CONSORT flow through one ‘wave’ of Club Connect

This was a single blind randomised controlled study design. Figure 1 illustrates the timeline of the assessment and intervention procedures. Participants were randomly allocated to either: immediate treatment (Club Connect), or b) a waitlist (control) group. This study was completed over three ‘waves’; the first wave was completed from February to May 2019, the second wave was completed from May to August 2019, and the third wave was completed from September to December 2019. This study was approved by St Vincent’s Hospital Human Research Ethics Committee (SVH 18/258) and all methods were performed in accordance with the relevant guidelines and regulations. The trial was registered on the Australian and New Zealand Clinical Trial Registry (ACTRN12619000195156, 12/02/2019). This study was supported by funding received from the St Vincent’s Clinic Foundation.

### Intervention

The intervention comprised ten weeks of either: (a) the Club Connect healthy brain ageing CT program; or (b) the waitlist control condition, as follows:

- Club Connect: as described in [45], comprised (i) 50 min of psychoeducation and (ii) 50 min of computer-based CT, with a 15–20 min break in between components where refreshments were provided and clinicians left the room (to facilitate peer socialisation).
  i. Psychoeducation: this component consisted of 10 semi-structured PowerPoint presentations on the following topics: the brain, attention and processing speed, learning and memory, executive functions, vascular risk factors, diet and exercise, depression and anxiety, and sleep. All material was delivered by multidisciplinary specialists (including Clinical Neuropsychologists, Clinical Psychologists, Occupational Therapists, and Old Age Psychiatrists) in the relevant field and in accordance with scientific literature, and a print out of all material was provided to participants. This component had a maximum of 14 participants per group which was based on our pilot data [49, 50] as well as data from other group-based CT interventions for older adults [45–47]; there was no predetermined minimum number of participants per group.
  ii. CT: the CT intervention was delivered primarily by Clinical Neuropsychologists, although Clinical Psychologists and Occupational Therapists provided assistance, and all utilised Neuropsychological Educational Approach to Cognitive Remediation (NEAR) which, by definition, entails group-based highly-individualised learning, and identifies three strategy types: restorative (which directly repairs cognitive skills via drill and practice), compensatory (where an individual learns to circumvent impaired cognitive skills) and environmental (where an individual considers changes to their environment to facilitate optimal cognitive functioning) [17]. In each session, participants undertook an individualised computer-based training program (comprising educational software and commercially available CT programs) devised according to their neuropsychological strengths and weaknesses (from baseline assessment). This component had a maximum of seven participants per group.
- Control: this included a waitlist period that was matched with clinician contact (with a weekly phone call). All participants were also sent a weekly letter via mail that provided a ‘healthy brain ageing’ tip developed by the research team, targeting a similar array of topics as those covered in the Club Connect psychoeducation sessions.

Given there is no available data regarding the appropriate ‘dose’ of CT for older adults with major depression, and in other patient cohorts, CT regimes differ significantly, the ‘dose’ utilised here was determined by CT experts (SN, LM), together with an expert Old Age Psychiatrist (LN) based on prior experience with delivering the HBA program to older adults.

### Randomisation

Participants were randomised to immediate treatment or waitlist conditions on a 1:1 basis using REDCAP software. The REDCAP Randomisation Module allows you to implement a pre-defined randomisation model, using an uploaded csv file (“allocation table”) where REDCAP will look up and find group assignments. The allocation table was created by a REDCAP team member, and was stored on a password protected server and concealed from both participants and researchers. Participants received their randomisation outcome after baseline assessments were completed. For ethical reasons, after completion of the follow-up assessments (i.e. after week 14), all control participants were offered the opportunity to complete the Club Connect program in the next available intervention group.

### Measures

All baseline assessments were conducted within a fortnight of commencing the intervention period (i.e. weeks one and two) and all blinded, follow-up assessments were conducted within a fortnight of completing the intervention period (i.e. weeks 13 and 14).

### Psychiatric and medical assessment

An Old Age Psychiatrist completed a full medical history, recorded depressive symptoms using the 17-item Hamilton Depression Rating Scale (HAM-D) [53], and assessed medical burden using the Cumulative Illness Rating Scale – Geriatric version [51].

### Neuropsychological assessment

A Clinical Neuropsychologist administered a battery of standardised neuropsychological tasks (listed below). Where possible, alternate forms were utilised and counter-balanced across baseline and follow-up assessments. Standardised scores (i.e. z-scores or age scaled scores) were calculated for all tests.

## Outcomes

### Primary outcomes

The primary outcome of this study was feasibility, defined as:

- Feasibility, or ‘reach’: including recruitment rates and/or strategy i.e. number/percent of referrals eligible and consented; conservative expected value of 50% (95% confidence interval, CI: 42–58%) based on a similar feasibility study with older adults [54].
- Tolerability, or ‘implementation’: adherence to treatment protocol for those randomised to the intervention group i.e. number/percent of sessions attended over a 10-week intervention period and number/percent of participants who completed baseline and follow-up assessments; expected value 66% (95% CI: 58–74%). Our “stop-go” measures were related to the proportion adhering - ≥66% - go to main trial, 50–65% – consider a modified trial design to increase adherence, if < 50% - do not progress to main trial using this model. Again, these expectations were based on a similar feasibility study with older adults [54].
- Acceptability, or ‘adoption’: of the Club Connect program, as perceived by clinical staff (who participated in focus groups) and patients randomised to the intervention (who completed a Club Connect patient experience survey). This data was used in exploratory qualitative analysis.
- Exploratory clinical outcomes to inform ‘effectiveness’ for a future trial: exploring the most appropriate and sensitive clinical outcome tools using effect sizes (and 95% confidence intervals) to inform appropriate sample size for an adequately powered definitive trial evaluation.

### Secondary outcomes: clinical

The secondary outcomes were intended to capture effect size change on expected measures of ‘effectiveness’ that might be considered for a full-scale trial. The below measures were administered to all participants (except where otherwise stated). They included:

- Neuropsychological functioning (for all tests below, higher standardised scores denote better performance).
  - Verbal learning and memory:
  i. The Hopkins Auditory Verbal Learning Test (HVLT) [55] was administered to measure unstructured verbal learning and recall. It comprises a list of 12 words. Total learning over three trials (maximum = 36) and delayed recall (maximum = 12) was examined. Alternate forms were available for this test and were counterbalanced.
  ii. The Story subtest from the Repeatable Battery for the Assessment of Neuropsychological Status (RBANS) [56] was used to measure structured verbal learning (maximum = 24) and recall (maximum = 12). Alternate forms were available for this test and were counterbalanced.
  - Visuospatial function and visual memory: Figure Copy and Figure Recall from the RBANS [56], was used to measure visuospatial function (maximum = 20) and visual memory (maximum = 20), respectively. Alternate forms were available for this test and were counterbalanced.
  - Language: the Controlled Oral Word Association Test (COWAT) [57] was used to measure generativity using letter (F, A, S) and semantic (types of animals) fluency, comprising the total number of words generated in three minutes and one minute, respectively. Alternate forms of letter (C, F, L) and semantic (types of fruits and vegetables) fluency were used and counterbalanced.
  - Speed: the Trail Making Test Part A (TMT-A, seconds) [58] was used to assess psychomotor speed.
  - Executive function: The Trail Making Test Part B (TMT-B, seconds) [58] was used to assess cognitive flexibility and the Delis–Kaplan Executive Function System (D-KEEFS) [59] Colour Word Interference Test was used to measure inhibition.

- Mood
  - Depression Anxiety and Stress Scale (DASS-21) [60] is a set of three self-report scales designed to measure the emotional states of depression, anxiety and stress, which are calculated by summing the scores for the relevant items. Higher scores indicate greater severity of symptoms.
  - Patient Health Questionnaire (PHQ-9) [61] is a self-report (i.e. subjective) instrument given to patients in a primary care setting to screen for the presence and severity of depression. The total sum of the responses suggests varying levels of depression and range from 0 to 27. In general, a total of 10 or above is suggestive of the presence of depression. The PHQ-9 is also used to evaluate efficacy of treatments for depression. A change of PHQ-9 score to less than 10 is considered a “partial response” to treatment and a change of PHQ-9 score to less than 5 is considered to be indicative of “remission.”
  - HAM-D [53] is a multiple item questionnaire used to measure of depression. It is rated by a clinician (i.e. objective measure of depression) on 17 items which are scored either on a 3-point or 5-point Likert-type scale. For the 17-item version, a score of 0–7 is considered to be normal, while a score of 20 or higher indicates at least moderate severity of depression.
- Sleep: Pittsburgh Sleep Quality Index (PSQI) [62] was used to measure sleep disturbance. Higher total scores (range 0–21) indicate poorer sleep quality.
- Quality of life: World Health Organisation Quality of Life Index (WHO-QoL) [63] is a self-report questionnaire that assesses four domains of quality of life: physical health, psychological health, social relationships, and environment. Higher domain scores denote higher quality of life.
- Wellbeing: WHO Wellbeing Index [64] was used to measure wellbeing. Higher scores indicate greater wellbeing.
- Functioning: clinician-rated functioning was measured using the Lawton Instrumental Activities of Daily Living Scale [65]. This measure is specific to older adults and examines instrumental activities including telephone use, shopping, food preparation, housekeeping, laundry, mode of transportation, responsibility for own medications and ability to handle finances. Higher scores indicate greater functional independence.
- Cognitive complaints: The British Columbia Cognitive Complaints Inventory (BC-CCI) [66] is a screening tool that assesses perceived cognitive difficulties specifically in patients with MDD and related mood disorders. Higher scores indicate more severe cognitive complaints.
- Service delivery: at follow-up, a patient experience evaluation form was administered to those randomised to the Club Connect intervention to gauge participant satisfaction of psychoeducation and computer-based CT.

### Secondary outcomes: healthcare resource use

We measured the resources used (costs) at key stages (and based on the RE-AIM domains) of the implementation of Club Connect. It is important to note that the classical RE-AIM model does not explicitly incorporate all the key elements of economic evaluation. However, it is widely acknowledged in the RE-AIM literature that resource use (cost) issues should be addressed in implementation studies [67–69]. The starting point for economic evaluation is identifying, calculating, and valuing all pertinent resources used (costs) [70].

In order to address some of the economic issues noted in [67] and others more recently in the literature [34, 36, 68, 69], we explicitly measured and valued applicable resource use (costs) required at different stages to deliver Club Connect and the waiting list control to assist health care decision-makers in determining resource allocation (e.g. for further scalability and transferability).

### Statistical analyses

Statistical analyses were performed using SPSS. Baseline data was summarised using means (with standard deviations), counts and proportions. The proportion of appropriate referrals of eligible and consenting participants (i.e. feasibility), and the adherence to the treatment protocol (i.e. tolerability) was calculated. In relation to acceptability data, interview transcript and free-text survey data from staff focus groups and patient experience surveys were used to explore acceptability and analysed following an iterative thematic analysis coding approach, which largely followed that of Braun and Clarke [71]. Independent research personnel (with expertise in qualitative research methods) used both inductive and deductive coding approaches on the data utilising the RE-AIM framework [67]. One research assistant facilitated and transcribed the focus group, and a different research assistant (as the first research assistant was subsequently unavailable) analysed the data. First, an inductive coding approach was used to address three specific research questions; (1) what were the aspects of the program that were perceived as particularly suitable to the target population, (2) what were the perceived benefits of program participation to staff and patients, and (3) what challenges, barriers and future improvements were identified by staff and patient participants of the program. Each focus group had approximately six clinicians and one facilitator. The facilitator was given a list of questions addressing the aforementioned themes and each clinician was given an opportunity to contribute. Second, a deductive coding approach was undertaken on the data to identify the domains and constructs of the RE-AIM framework [67] which were implicated by staff participants in the facilitation of the intervention.

For each clinical outcome measure, a two-way repeated measures ANOVA with effect sizes (i.e. partial eta squared) was constructed. Analyses were tested for a Condition x Time interaction to explore differences in participants’ performance on all clinical outcomes (i.e. measures of cognition, mood, etc.) before and after the 10-week intervention. All analyses were two-tailed and used an alpha value of 0.05.

## Results

### Descriptive sample characteristics

Forty participants were enrolled in Club Connect, comprising three waves of recruitment, data collection and intervention delivery. Twenty-one participants were randomised to the Club Connect intervention and 19 participants were randomised to the waiting list control (see Table 1). Across groups, the average age of participants was 76.13 years (SD: 7.45, range: 65–95 years) and 70% were female. The cohort had 13.89 years (SD: 3.62, range: 6–23) of formal education on average and the mean standard score on the Test of Premorbid Functioning was 108 (SD: 13.40, range: 85–125), indicating an average level of estimated premorbid intellectual functioning. The mean MMSE score was within normal limits, i.e. 27.5 (SD: 1.96, range: 23–30), and 78% reported English as their first language. The mean score on the GDS-15 was 7.15 (SD: 3.46, range: 1–15), and 38% (or 14/36) of participants were taking at least one psychotropic medication. A series of independent t-tests demonstrated that there was no statistical difference between the Club Connect group and the waiting list control group at baseline (see Table 1).

**Table 1:** Descriptive baseline characteristics

	Club Connect		Control		Total		*p* value
	N	M(SD)	N	M(SD)	N	M(SD)	
Age	21	76.24(8.13)	19	76.00(6.83)	40	76.13(7.45)	0.92
Sex, n, %	21	16, 76%	19	12, 63%	40	28, 70%	0.88
Education	21	14.45(3.27)	19	13.26(3.66)	40	13.89(3.62)	1.04
TOPF	11	109.64(10.30)	10	106.30(16.57)	21	108.05(13.40)	0.58
MMSE	21	27.71(1.82)	19	27.26(2.13)	40	27.50(1.96)	0.48
GDS-15	21	6.57(3.49)	19	7.79(3.41)	40	7.15(3.46)	0.27

GDS-15 = Geriatric Depression Scale, 15-item; M = mean; MMSE = Mini Mental State Examination; SD = standard deviation; *p* = comparison between two groups; TOPF = Test of Premorbid Functioning

### Feasibility

Sixty-one participants were screened for Club Connect. The expected recruitment rate of eligible and consented participants to indicate feasibility or ‘reach’ was 50%, but the actual percentage of eligible referrals recruited exceeded this at 84% (51 participants), as did the percentage of those consented and enrolled at 78% (40 participants). Therefore, this study was considered feasible. A series of independent t-tests demonstrated that there was no statistical difference between completers and non-completers at baseline on the following variables: age (*t* [37] = 2.02, *p* = 0.05), education (*t* [37] = 0.13, *p* = 0.90), MMSE (*t* [37]=-0.53, *p* = 0.60) and depression symptomatology (*t* [37]=-1.16, *p* = 0.25).

The primary reasons that eligible individuals were not enrolled at baseline were:  (a) becoming unwell, being overcommitted, or an unknown reason (n = 8), (b) failing to attend baseline assessment (n = 2), or, (c) no longer meeting eligibility criteria (n = 1).

### Tolerability

In terms of tolerability and session attendance, the percentage of participants randomised to the intervention group (21 participants) that attended seven or more sessions was 81% (17/21 participants), exceeding the expected value of 66%. In terms of session attendance, of the 21 participants randomised to Club Connect, one participant withdrew before commencement of the group due to deterioration in mental health, and a further two participants each attended three sessions before withdrawing due to deterioration in physical health for one and deterioration in mental health for the other (although the former individual did complete follow-up assessment). Of the remaining 18 participants in the intervention group, 89% attended ≥ 9 of the 10 sessions, with a mean attendance rate of 9.38 (range: 6–10). Importantly, there were no adverse events reported by participants.

The expected rate of data collection was 66%, but the actual percentage of completed data collection was 100% at baseline and 90% (36 participants) at follow-up (i.e. attended both baseline and follow-up assessment). The reasons participants did not complete data collection at follow-up were: (a) a deterioration in mental health (n = 2, as above, both were randomised to Club Connect), or being overcommitted (n = 1, randomised to waitlist control), or (b) pre-arranged travel (n = 1, randomised to waitlist control).

### Acceptability

All those participants who were randomised to Club Connect and completed the program (n = 19/21) anonymously completed the patient experience survey, although some participants did not respond to all items. All respondents (100%, 19/19) agreed that getting to St Vincent’s Hospital was not too difficult; that the 10-week program was manageable; that there was enough and easy access to support from clinicians between sessions; that the clinicians were knowledgeable on topics related to HBA; that the lecture material was useful; and, that they enjoyed being part of a group and benefitted from the social component of Club Connect. The large majority (95%, n = 18/19) agreed that the computer training was enjoyable and useful, and not too difficult. Only one participant (5%, n = 1/19) indicated that the lecture material was not relevant or useful for them. The large majority (95%, n = 18/19) agreed that the assessment and data collection process was not too long or difficult. Two participants (11%, n = 2/18) indicated that their physical health was a barrier to participating in Club Connect. All respondents indicated that they would recommend participating in Club Connect to others.

Focus group data indicated a general sentiment that the program was well accepted among staff participants. Specifically, qualitative analyses revealed that this was due to: (a) the intervention being suitable for the target group in that it was individually tailored and practically suitable (i.e. centrally located, close to public transport, appropriate in duration, small group size etc.), (b) the structural characteristics of the program (i.e. embedding the program within a multidisciplinary team), (c) the strong communication within the Older People’s Mental Health Service (i.e. strong implementation leader or champion within the service), (d), the implementation climate (i.e. Older People’s Mental Health Service being perceived as innovative), and (e) knowledge-beliefs in regard to the intervention being evidence-based.

In addition, while our focus here was acceptability, or ‘adoption’ of the intervention, as perceived by staff and patients, there were several other aspects of the program obtained during exploratory qualitative data collection and analysis that were identified as being important in facilitating the intervention. These facilitators are summarised in terms of the five major domains of the RE-AIM framework (see Table 2). Further, staff and patient participants described challenges and barriers to participation in Club Connect, which together with suggested future improvements, are described by participants (see Table 3).

**Table 2:** Facilitators to program implementation identified by staff and patient participants, grouped by RE-AIM domains and constructs, with quotes

RE-AIM domains	Level	Constructs identified	Example quotes
Reach	Individual	• **Suitable to target group** i. multi-disciplinary facilitation was perceived by staff to maintain patient interest in and attention to, the lecture content ii. individually tailored strategies e.g. tailored computer games, written material for patients to re-visit iii. social rituals (i.e. tea break) iv. practical aspects of the program: small group size, being centrally located and/or close to public transport, appropriate in duration	“…*the computer games were fun and sometimes challenging. On the other hand, we could play the computer games at a level which suited each individual” [patient 2]* “*I actually think that that (the tea time) is one of the most valuable parts of the program” P11, “I don’t think you can negate the benefits of that period in between that allowed them to engage with each other” P10* *“It’s a very targeted program. Keeping very much the patient right at the forefront of the design” P3* *“…they get a booklet […] they can read through the notes in advance, have the lecture and also revise it afterwards […] it helps to overcome maybe some of the challenges that are associated with cognitive impairment” P4* *“small group of people was good for me because of my hearing loss” [patient 3]* *“close to public transport” [patient 1], “proximity and being close to [the hospital]” [patient 2]* *“it doesn’t go for too long […] I think it is well targeted to our population and our age group and therefore the cognitive impairment” [P10]*
Effectiveness	Individual	• **Improved job satisfaction** i. professionally rewarding ii. meets clinical ‘need’ • **Positive outcomes for patients** i. more engaged generally ii. increased social connections iii. normalised patient experience a. cognitive impairment b. low mood • **Intervention source** i. staff perceived the intervention to be internally developed and driven • **Evidence strength and quality** i. staff perceived the intervention to be evidence-based	*“I think professionally, that it is a really rewarding thing to be involved in” [P3]* *“in the absence of any medications to cure cognitive impairment and dementia, you know people are wanting something that they can do” [P10]* *“I think… being involved in Club Connect meant they… want(ed) to kind of improve, I think I did actually notice that those consumers that I worked with [independent to the group] were quite motivated and… engaged in [psychological] therapy” [P4].* “*A lot of the referrals (are) for (those) who (are) socially isolated or have limited social activities. Put these different clients in a room and you add to that social experience - you see these people who maybe once were depressed or socially isolated start to communicate with their peers” [P6];* “*I have been very lonely for a long time, and more and more just by myself. Through Club Connect’s encouragement […] I’m now in a much improved mental and physical health” [patient 3]* *“our cohort still carry a certain amount of stigma or shame, and it’s amazing how you see this group really come together and normalise and validate one another’s experiences” [P11], "[Club Connect] opened my mind. [I’m] not afraid of getting old" [patient 5]* *“The fact that it was embedded within the team is an important thing. It’s not some project that sits outside the critical day to day function”[P7]* *“There’s a lot of evidence to suggest that this kind of cognitive remediation works well in certain research populations” [P3]*
Adoption	Organisation	• **Networks and communications** i. regular team discussions and ‘check-ins’ ii. easy access for staff to liaise with facilitators for referrals • **Structural characteristics** i. embedding program within multi-disciplinary team allows for multi-disciplinary facilitation ii. implementation leader: having a clinician on the team to lead or champion the project • **Implementation climate** i. team considered to be innovative • **Knowledge-beliefs about intervention** i. team members were knowledgeable about the program ii. team members believed the program would impact change	*“…because we were all on the same team and we maybe share patients and talk to each other about all those people every week. It was easy [to identify people for program]” [P7]* *“Ease of making a referral to Club Connect, ability to discuss with Club Connect providers about the eligibility criteria and about specific cases to see if they would benefit from Club Connect” [P13]* *“The biggest enabler is actually having the facilitator as part of the team. She’s there all the time. So, whenever you have someone come up that you think might be suitable for the group, you just run it by her and she kind of facilitates that referral” [P11]* *“… because of the multi-disciplinary nature of team, you know we can, for example, with the education sessions, have different specialists come in and out quite flexibly” [P11]* *“as a team, I think we’re quite an innovative team. I think we’re open to the idea of trying new projects, developing new programs” [P11]* *“It’s high knowledge. Everybody on the team knows about the program” [P5]* *“It’s also really good to see group programs now that there’s a lot of evidence to suggest that this kind of cognitive remediation works well” [P3]* *“I think professionally, [the program] is a really rewarding thing to be involved in” [P3]* *“… you sit with [patients] each week, you work together and you see a level of confidence and skill build, and it’s a pleasure” [P11]* *“But I think this is testament to the idea that if you have champions of these programs, that it is achievable. It becomes just a fluid part of what you do, I think particularly when you’ve got dedicated people looking after it”[P11]*
Implementation	Organisation	• **Adaptability** i. perceived by staff to be easily adaptable to include patients with other needs (e.g. translation into languages other than English, adapting materials for vision or hearing impaired participants) ii. strategies used to simplify execution, e.g. materials / scripts provided to facilitators to not only enrol patients in program, but also to facilitate sessions	*“I think it’s a program that could be sort of readily modified and adapted to an array of different populations where themes of cognition, social isolation, etcetera are prevalent issues” [P11]* *“… [for enrolling patients] they had a really good script where […] you can just come in and you know exactly what you need to ask” [P12]*
Maintenance	Organisation	• No follow-up, therefore no data collected on ‘Maintenance’.	

*P* = staff participant

**Table 3:** Challenges and barriers to program participation, and suggested future improvements, identified by staff and patient participants

**Challenges for staff:**
• Organisational aspects of a multiple-staff intervention (e.g. having enough staff to: facilitate different lecture topics, run computer training, or step in if someone is unavailable) • Time commitment to help facilitate the intervention, and to complete paperwork • Managing interpersonal difficulties amongst the patient group
**Barriers to patients participating in program**:
• Access to transport • Wait time for groups to start • Out of catchment area referrals
**Suggested improvements**:
• Having a dedicated person on team who manages program administration duties • Providing psychoeducation (verbal or written) about the group-based format to patients prior to commencing the program, to ease any anxiety • Organising transport options for patients to attend the program • Embedding the program as a permanent part of the service, so that the timing of groups is predictable • Expanding program participation to involve carers • Ensuring ongoing links to other social groups in the community to ‘fill the gap’ once the program ends • Modifying the program content for other populations (vision or hearing impaired, languages other than English)

### Data analysis of secondary clinical outcomes

Several two-way repeated measures ANOVAs were conducted to determine the magnitude of change on secondary clinical outcomes at baseline and follow-up following randomisation to the intervention group or the control group (see Table 4). Results demonstrated a significant interaction effect, whereby treatment was associated with large to very large effect size improvements for depressive symptoms on the HAM-D, *F* [1, 25] = 4.994, *p* = 0.035, ηp^2^ = 0.172) and for a measure of inhibition from the DKEFS, *F* [1, 33] = 8.715, *p* = 0.006, ηp^2^ = 0.209), and a moderate effect size improvement on the Stress scale of the DASS *F* [1, 34] = 4.410, *p* = 0.043, ηp^2^ = 0.115). In addition, medium effect size improvements were evident on the Depression scale of the DASS, on a measure of instrumental ADLs and on the WHO Wellbeing scale, as well as cognitive outcomes including verbal learning (HVLT learning) and one measure of executive function (Trails B). Small effect size improvements were evident on other cognitive and psychosocial outcomes (see Table 4). Missing neuropsychological and psychosocial data was not controlled for statistically.

**Table 4:** Baseline and follow-up neuropsychological, mood and psychosocial data

	Club Connect			Control			Overall	
Outcome	N	Mean(SD) pre-intervention	Mean(SD) post-intervention	N	Mean(SD) pre-intervention	Mean(SD) post- intervention	*F*, *p*-value	Partial Eta Squared
HVLT total learning z-score ^a^	19	-0.06(1.31)	0.96(2.07)	16	-0.26(1.14)	-0.13(1.59)	2.038, 0.163	**0.058** ^**++**^
HVLT delay z-score ^a^	18	-0.14(1.29)	0.03(1.28)	16	-1.02(4.70)	-0.12(0.92)	0.443, 0.510	0.014^+^
HVLT DI z-score ^a^	18	0.19(1.03)	0.26(0.95)	16	0.12(0.86)	-0.03(0.96)	0.680, 0.416	0.021^+^
RBANS Story I SS ^b^	19	10.79(2.39)	11.68(2.71)	16	10.50(2.42)	11.23(2.87)	0.439, 0.512	0.013^+^
RBANS Story II SS ^b^	19	10.84(2.65)	11.21(2.49)	16	10.25(2.44)	9.75(2.11)	1.225, 0.276	0.036^+^
RBANS Figure I SS ^b^	19	11.11(2.47)	8.16(2.97)	16	10.69(3.00)	9.00(2.82)	1.958, 0.171	**0.056** ^**++**^
RBANS Figure II SS ^b^	19	9.05(2.74)	8.53(2.29)	16	8.69(3.07)	8.88(1.82)	0.692, 0.412	0.021^+^
Letter fluency total z-score ^a^	19	0.32(1.25)	0.35(0.97)	16	0.19(1.65)	0.06(1.59)	0.406, 0.529	0.012^+^
Animal fluency z-score ^a^	19	0.37(1.36)	0.12(1.12)	16	-0.43(1.40)	0.07(1.43)	3.620, 0.066	**0.099** ^**++**^
Trails A z-score ^a^	19	0.26(1.01)	0.50(0.99)	16	-0.75(1.85)	-0.54(1.63)	0.000, 0.996	0.000
Trails B z-score ^a^	18	-1.11(2.76)	-0.61(1.64)	12	-0.11(0.97)	-0.74(1.95)	3.398, 0.076	**0.108** ^**++**^
Colour Word Interference Test Naming SS ^c^	19	9.58(3.61)	10.37(2.83)	16	8.38(4.05)	8.31(4.22)	1.155, 0.290	0.034^+^
Colour Word Interference Test Reading SS ^c^	19	10.63(2.77)	10.74(2.58)	16	11.0(9.52)	9.13(3.69)	0.939, 0.340	0.028^+^
Colour Word Interference Test Inhibition SS ^c^	19	10.37(3.64)	11.63(2.75)	16	9.31(4.05)	8.75(4.55)	8.715, 0.006	**0.209** ^**+++**^
Colour Word Interference Test Inhibition Switching SS ^c^	19	12.68(8.35)	11.26(2.33)	14	8.79(3.95)	9.93(3.83)	1.378, 0.249	**0.043** ^**++**^
PhQ-9 ^d^	18	8.17(6.94)	6.06(4.33)	15	8.73(4.53)	8.7(7.04)	0.770, 0.387	0.024^+^
HAM-D ^d^	15	9.13(4.8)	7.87(4.32)	11	8.36(5.24)	12.18(7.28)	4.994, 0.035	**0.172** ^**+++**^
DASS Depression ^d^	19	5.80(5.54)	4.05(3.22)	17	5.53(3.39)	6.40(5.4)	2.536, 0.121	**0.069** ^**++**^
DASS Anxiety ^d^	19	2.80(2.80)	2.26(1.79)	17	3.24(2.19)	2.65(2.57)	0.007, 0.933	0.000
DASS Stress ^d^	19	5.90(4.97)	3.53(2.93)	17	3.82(3.19)	4.18(3.89)	4.410, 0.043	**0.115** ^**+++**^
iADL ^d^	18	7.50(0.92)	7.89(0.32)	17	7.24(1.3)	7.35(1.32)	2.441, 0.127	.**067**^**++**^
PSQI ^d^	19	1.79(1.13)	1.53(1.17)	17	1.30(1.36)	1.47(1.37)	0.485, 0.491	0.014^+^
BC-CCI ^d^	19	7.05(4.16)	6.47(4.30)	17	9.59(5.03)	9.53(6.76)	0.226, 0.638	0.007
WHO Wellbeing ^d^	19	13.05(5.21)	15.00(5.43)	17	12.59(5.32)	11.82(6.84)	2.482, 0.124	**0.068** ^**++**^
WHO-QoL Physical ^d^	19	281.02(43.47)	293.05(35.26)	17	285.08(51.45)	285.08(48.91)	0.603, 0.443	0.017^+^
WHO-QoL Psychological ^d^	19	275.88(50.76)	283.77(41.27)	17	274.02(39.30)	268.14(41.27)	1.728, 0.197	**0.048** ^**++**^
WHO-QoL Social ^d^	19	311.84(72.77)	303.07(84.81)	17	298.53(85.61)	300.49(93.93)	0.357, 0.554	0.010^+^
WHO-QoL Environment ^d^	19	350.66(49.73)	351.32(49.46)	17	354.41(49.79)	342.65(66.74)	0.842, 0.365	0.024^+^

^a^ age and educated corrected z-score; ^b^ age and educated corrected scaled score; ^c^ age corrected scaled score; ^d^ total raw score; ^+^ indicates small effect size (Hedges’ g < 0.3); ^++^ indicates medium effect size (Hedges’ g = 0.3–0.6); ^+++^ indicates large to very large effect size (Hedges’ g > 0.6); BC-CCI = British Columbia Cognitive Complaints Inventory; DASS = Depression Anxiety Stress Scale; DI = discrimination index; HAM-D: Hamilton Depression Rating Scale; HVLT = Hopkins Auditory Verbal Learning Test; iADL = Instrumental Activities of Daily Living Scale; PhQ-9 = Patient Health Questionairre-9; PSQI = Pittsburgh Sleep Quality Index; RBANS = Repeatable Battery for the Assessment of Neuropsychological Status; SS = scaled score; Qol = Quality of Life; WHO = World Health Organisation

### Healthcare resource use

Table 5 provides estimates of the resources used (costs) for the Club Connect intervention from the viewpoint of a healthcare provider. The cost calculations were based on a sample of *n* = 8, the number of people who would typically complete one ‘wave’ of the Club Connect intervention based on pilot data [49, 50]. The largest costs incurred were the time costs of the clinician for assessment and delivery of the Club Connect intervention (totalling AU$2,644 or 46.62%). This was closely followed by the costs required to set-up the computer lab (totalling AU$6,400), although it was anticipated that the computers would be used by (at least) 24 participants over a 12-month period, therefore costing AU$2,133.60 or 43.93% for one ‘wave’ of Club Connect (*n* = 8). The total cost of delivering one wave (*n* = 8) of Club Connect was therefore calculated to be AU$4,856.70 and the average cost per person was calculated as AU$607.50.

**Table 5:** Healthcare provider and patients’ costs associated with one ‘wave’ of Club Connect (n = 8 patients) ^a^

Item	Description	Cost Calculation	Total Costs
Clinician time for brief neuropsychological assessment (required for individualised CT program)	45 min neuropsychologist time per patient	$45.10 per assessment based on $60.16 per hour for clinical psychologist services^1^	$360.80
Clinician (multidisciplinary) time for delivery of the intervention	60 min psychoeducation per week for 10 weeks + 60 min for 2 clinicians to facilitate computer-based CT for 10 weeks	$70 per hour based on mean hourly rate of clinical psychologist services^1^, psychiatry registrar and old age psychiatrist^2^ × 10 weeks + $60.16 per hour based on clinical psychologist services^1^ × 2 clinicians x 10 weeks	$1,903.20
Club Connect workbook	Printing and binding per workbook	$17.40 for each participant	$139.20
Computer lab costs for computer-based CT	8 desktop computers	$800 for each computer =$6,400; used by 24 participants over 12-month period, amounting to $266.70 per participant	$2,133.60
Refreshments	Arnott’s biscuits 3 kg + tea, coffee, sugar	$43.00	$43.00
Administration costs for organisation of baseline assessment and follow-up of group attendance each week	Estimate 2 hours for baseline assessments and 30 min per week for follow-up of group attendance	$279.90 based on $39.98 per hour for psychological services^1^	$279.90
Total for intervention Average cost per participant			$4,856.70 $607.50

^a^ n = 8 is the number of participants who would typically complete one ‘wave’ of the Club Connect intervention, and thus we assume 8 participants in the control arm as well

^1^ = NSW Health Psychology (State) Award; ^2^ = NSW Health Staff Specialist (State) Award

CT = cognitive training; OPMH = Older People’s Mental Health

## Discussion

This is the first known study to implement an evidence-based CT program for older adults with MDD in a clinical, ‘real-world’ setting. We demonstrate, for the first time, feasibility (‘reach’), tolerability (‘implementation’) and acceptability (‘adoption’) for translating group-based CT into clinical practice.

Primary outcomes for this trial related to feasibility, implementation and acceptability. Regarding the former, given that 84% of referred participants met eligibility criteria, and 78% were enrolled, which exceeded our *a priori* estimate of 50%, the overall trial design was considered feasible in that we were able to ‘reach’ appropriate patients. Overall, our recruitment rate is slightly higher than in most other studies of CT implementation in schizophrenia or psychosis, where about 47–66% of participants are reported to be recruited [34, 37, 72], and about 45–62% are reported to be enrolled [34, 37, 72]. However, interestingly, our recruitment and enrolment figures are similar to another trial that specifically targeted depressed outpatients [39], which perhaps suggests that this type of intervention is particularly suited to older adult outpatients with affective conditions.

In terms of implementation, session attendance rates demonstrated that 81% of participants randomised to the intervention group attended seven or more sessions, which exceeded our *a priori* estimate of 66%, thus indicating the intervention was ‘tolerable’. Although limited data is available on session attendance across other studies of CT implementation, available data indicates mean attendance rates between 70 and 84% [36, 39], which is largely consistent with our results. In terms of tolerability of data collection procedures, given that the percentage of completed baseline and follow-up data collection was 100% and 90%, respectively, exceeding our *a priori* estimate of 66%, the data collection process was similarly considered ‘tolerable’. Again, our results are relatively consistent with other CT implementation studies, where data collection and study completion rates of 66% [36] and 73% [72] are reported, in those with either a mood disorder or schizophrenia, or in those with schizophrenia, respectively, and 95% in those with depression [39]. Further, in terms of tolerability, the current trial is notable for the absence of any reported adverse events, unlike pharmacological interventions for major depression [73].

In regard to acceptability or ‘adoption’, 100% of participants who were randomised and completed Club Connect anonymously reported that they would recommend the program to their peers, demonstrating overwhelming acceptability of the program. Qualitative feedback from participants and staff indicated a general sentiment that the intervention was suitable for the target group; facilitated increased social connections among patients; normalised the patient experience; and facilitated greater patient engagement. Staff reported improved job satisfaction and appreciated the importance of several contextual factors (e.g. networks and communications, leadership, the implementation climate, knowledge-beliefs about the intervention etc.).

As a secondary outcome, we sought to explore direction and size of effects on expected measures of ‘effectiveness’ that might be considered for a full-scale trial. We found that Club Connect was associated with large effect size improvements for clinician-rated depression and on an objective test of executive function (i.e. inhibition), and a moderate effect size improvement on self-rated stress. In addition, moderate effect size improvements were evident for instrumental ADLs and wellbeing, and cognitively, on a verbal learning task and another test of executive function (i.e. cognitive flexibility). These preliminary results are generally consistent with our prior work [30, 45, 74], although the reported improvement in instrumental ADLs is unique to the current trial, which is especially encouraging as this may represent generalisability or ‘far transfer’ of effects to functional outcomes. Nonetheless, while encouraging, this study was not powered to investigate efficacy and therefore it is difficult to make firm conclusions regarding the clinical benefits or dose effects of CT here. Notwithstanding this caveat, evidence from these validated measures suggest, at the very least, that outcome measurement tools were sufficiently sensitive to detect change post-intervention and therefore we are now able to specify primary and secondary outcomes, and calculate power, for a definitive trial. To this end, analyses revealed that the mean difference of change in verbal memory (while controlling for improvement in mood) between the intervention and the control group was estimated at 1.35, with a pooled standardised mean difference of 2.5, suggesting a medium effect size (Cohen’s d = 0.5). Therefore, a sample size of 104 participants (52 per group) will be required to detect the effect size mean difference of 0.5 (assuming the correlation between pre and post measures is 0.6, with 80% power and two-sided level of significance at 0.05).

We also sought to measure the healthcare resources used (costs) associated with the Club Connect intervention. The two main cost categories under the intervention, from a healthcare provider viewpoint, were the time costs of health professionals in assessing and delivering the Club Connect intervention (summing AU $2,644 or 46.62%), and the set-up costs for the computer lab for computer-based CT (maintained at AU $2,133.60 or 43.93%). The average cost per participant was calculated to be $607.50 for one wave of Club Connect. Relative to other behavioural interventions for major depression, such as cognitive behavioural therapy and computer-assisted cognitive behavioural therapy, which have been costed at US $2,166 (AU $2,498) and US $1,247 (AU $1,439) per person [75], respectively, Club Connect not only has the potential to be a cost saving intervention (by as much as 60%), but directly also addresses neurocognitive impairment which other interventions for MDD do not.

Whilst this feasibility trial overall demonstrated successful implementation of the Club Connect program, we also recognise several limitations. First, we were not able to include longitudinal follow-up and therefore do not have data on ‘maintenance’ of intervention effects in these individuals and in this setting over time. This is a common methodological issue across the CT literature [76], potentially reflecting limits in funding and resource availability for clinical research; nonetheless, sustainability of effects is imperative to properly assess the utility of CT as a viable and effective intervention for neurocognitive deficits in depression within the clinical setting. Ideally, longitudinal follow-up may be incorporated into the next-step full-scale RCT to address this. Second, the contribution and severity of depressive symptoms, the cognitive status of participants, and concurrent psychosocial and pharmacological treatments, while evaluated (to some degree), were not controlled for in the current study (although there was no statistical difference between groups on these variables). Third, in relation to ‘effectiveness’, although we were not sufficiently powered to determine efficacy of CT within this setting, we did not statistically control for missing neuropsychological and psychosocial data (although there was no statistical difference at baseline on age, education, cognitive status and depressive symptomatology between completers and non-completers), and we did not control for any impact of socialisation on results. Fourth, in regard to healthcare resources used (costs), data on resource use from the viewpoint of patients and their families or friends assisting them with their care (such as costs to travel to and from the intervention site, costs incurred by carers for accompanying the patient to the intervention site i.e. time off from work and parking costs at the site) should be included in more large-scale studies to assess cost from a wider societal perspective. Fifth, in relation to ‘acceptability’, only one researcher analysed the thematic data (due to resourcing limitations). Finally, the primary focus of implementation was client-based; while service providers (or clinicians) were involved in study design and provided ongoing evaluation on implementation, input on implementation from the organisation or from a systems-level perspective was not obtained.

Future studies should not only address the above limitations, but also address a number of other unresolved issues in order to facilitate greater implementation of CT interventions into everyday clinical practice, including: the use and qualifications of program facilitators, and their training and supervision requirements; the target population, including diagnosis and/or the presence of cognitive and/or functional impairments, and the expertise required for the assessment of the aforementioned; program goals (cognitive vs. functional); financial support required to implement and facilitate such programs [36]; and the inclusion of quality-of-life measures to enable a broader assessment of the clinical benefits and cost-effectiveness.

In summary, this implementation study demonstrates feasibility (with 84% of referrals meeting eligibility criteria and 78% of those being enrolled), acceptability (with 100% of participating reporting they would recommend Club Connect) and tolerability (with 81% attending seven or more of the ten sessions) of a structured, group-based CT program among staff and among older adult patients with major depression within the clinical setting. Our results, in light of the recognised ‘evidence to practice gap’, of interventions for neurocognitive impairment in older adults with depression, highlights the potential for CT interventions to be adopted and embedded within clinical settings (especially given that these intervention may be cost-saving), and underscores the urgent need for further translational research evaluating the effectiveness of CT in everyday clinical practice.

## Data Availability

The datasets used and/or analysed during the current study are available from the corresponding author on reasonable request.

## List of abbreviations

ADLs: Activities of daily living
ANOVA: Analysis of variance
BC-CCI: British Columbia Cognitive Complaints Inventory
CI: Confidence interval
COWAT: Controlled Oral Word Association Test
CT: Cognitive training
DASS-21: Depression Anxiety Stress Scale, 21-item
D-KEFS: Delis–Kaplan Executive Function System
GDS-15: Geriatric Depression Scale, 15-item
HAM-D: Hamilton Depression Rating Scale
HVLT: Hopkins Auditory Verbal Learning Test
RBANS: Repeatable Battery for the Assessment of Neuropsychological Status
MCI: Mild Cognitive Impairment
MDD: Major Depressive Disorder
MMSE: Mini Mental State Examination
NEAR: Neuropsychological Educational Approach to Cognitive Remediation
PHQ-9: Patient Health Questionnaire
PSQI: Pittsburgh Sleep Quality Index
RCT: Randomised controlled trial
RE-AIM: Reach, effectiveness, adoption, implementation, maintenance
TMT-A: Trail Making Test Part A
TMT-B: Trail Making Test Part B
WHO-QoL: World Health Organisation Quality of Life Index
